# The inflammatory cytokine effect of Pam3CSK4 TLR2 agonist alone or in combination with *Leishmania infantum* antigen on ex-vivo whole blood from sick and resistant dogs

**DOI:** 10.1186/s13071-017-2062-3

**Published:** 2017-03-13

**Authors:** Pamela Martínez-Orellana, Paulina Quirola-Amores, Sara Montserrat-Sangrà, Laura Ordeix, Joan Llull, Alejandra Álvarez-Fernández, Laia Solano-Gallego

**Affiliations:** 1 Departament de Medicina i Cirurgia Animals, Facultat de Veterinària, Universitat Autònoma de Barcelona, Bellaterra, Barcelona, Spain; 2 Hospital Mon Veterinari, Manacor, Mallorca Spain; 3 Clinica Veterinaria Noreña, Noreña, Asturias Spain

**Keywords:** Ibizan hound, Inflammatory cytokines, *Leishmania infantum*, Sick dog, TLR-2 agonist (Pam3CSK4)

## Abstract

**Background:**

A wide spectrum of clinical manifestations and immune responses exist in canine *L. infantum* infection. Ibizan hounds are more “resistant” to disease than other dog breeds. Recognition of pathogen-associated molecule patterns by toll like receptors (TLRs) rapidly triggers a variety of anti-microbial immune responses through the induction of pro-inflammatory cytokines such as TNF-α and IL-6 which may play an important role in controlling *Leishmania* infection. The main objective of this study was to investigate and compare the effect of a TLR2 agonist (TLR2a) alone or in combination with *L. infantum* antigen (LSA) on ex vivo whole blood cytokine production from healthy seronegative IFN-γ non-producer dogs from an area of low in canine leishmaniosis endemicity (*n* = 11); sick seropositive dogs with low production of IFN-γ (*n* = 17) and healthy seronegative or low positive Ibizan hounds with a predominant IFN-γ production (*n* = 21) from a highly endemic area. Whole blood was stimulated with medium alone (Ø), LSA, concanavalin A, TLR2 (Pam3CSK4) receptor agonist (Ø + TLR2a) and TLR2a and LSA (LSA + TLR2a) for 48 h. Supernatants were harvested for measurement of canine TNF-α and IL-6 cytokines by ELISA.

**Results:**

A significant increase of TNF-α was found in the supernatants of stimulated blood from all groups (Ø + TLR2a and LSA + TLR2a) when compared with medium alone. A similar pattern was observed for IL-6. Interestingly, a significant increase of TNF-α production was only observed when stimulation with LSA + TLR2a was compared with TLR2a alone in Ibizan hounds. A significant increase of TNF-α production was observed with stimulation of LSA + TLR2a when compared with LSA in all groups. Significantly higher concentrations of TNF-α and IL-6 were detected in Ibizan hounds, especially for the Ø + TLR2a and LSA + TLR2a treatments compared with other groups.

**Conclusions:**

This study demonstrated that TLR2a alone enhances the production of the inflammatory cytokines TNF-α and IL-6 in sick, “resistant” and healthy non-infected dogs. In addition, a combination of LSA+TLR2a promoted a synergistic pro-inflammatory effect with TNF-α in Ibizan hounds but not in seropositive sick dogs and seronegative healthy dogs. These findings might suggest the importance of Pam3CSK4 as a possible immunomodulator for CanL.

**Electronic supplementary material:**

The online version of this article (doi:10.1186/s13071-017-2062-3) contains supplementary material, which is available to authorized users.

## Background

Canine leishmaniosis (CanL) due to *L. infantum* is a life-threatening sand fly-borne zoonotic disease with a wide distribution in Central and South America, Asia, Africa and the Mediterranean basin regions [1]. The seroprevalence for leishmaniosis reported in dogs in the Mediterranean basin ranges from 5 to 30% depending on the region [2]. A broad range of immune responses and several degrees of disease have been described for CanL ranging in severity from a chronic subclinical infection, a self-limiting disease, to non-self-limiting illness, determining the prognosis and treatment options [2, 3]. Therefore, a clinical staging system of this disease is currently used in the clinical setting [2].

The immune responses mounted by dogs at the time of infection and thereafter appear to be the most important factor in determining whether and when the infection will progress from a subclinical state into clinical illness [4]. Dogs that are able to control infection by either resolving it and eliminating the parasite or restricting the infection and remaining consistently subclinical are considered clinically “resistant” [3]. As an example, Ibizan hounds appear to be more “resistant” than other more susceptible dog breeds and rarely develop clinical signs related with *L. infantum* infection [5–7]. Therefore, this breed provides an interesting model to study the origin of this kind of apparently immunological resistance. In contrast, dog breeds that are predisposed to developing a disseminated infection and progressing towards clinical CanL are considered susceptible (e.g., Boxer, Cocker Spaniel, Rottweiler and German Shepherd) [7].

The ability of the host to control *L. infantum* infection requires the generation of cellular mediated immune (CMI) responses, which activate host infected macrophages in order to kill intracellular *Leishmania* parasites [8]. CMI protection is given by the activation of CD4+ T helper cells (Th) from the adaptive immunity, which is influenced by a mixed response due to the balance between Th1-like lymphocytes (Th1) and Th2-like lymphocytes (Th2) [8]. Classically, the polarized Th1/Th2 (pro-inflammatory/anti-inflammatory) response against *Leishmania* infection was associated with rodent models [9]. On the other hand, humans and dogs seem to develop a more intricate and complex immune response. Previous studies performed on peripheral blood samples from *Leishmania*-infected dogs described a protective induction of a predominant Th1 response that was associated with the activation of cells, producing IFN-γ, IL-2 and TNF-α, that was correlated with immunity and healing [10]. After activation mediated by IFN-γ, macrophages produce TNF-α that increases reactive oxygen substances (ROS) in peripheral blood mononuclear cells (PBMCs) from dogs with leishmaniosis leading *Leishmania* destruction [11]. This T-cell activation constitutes the cornerstone and the link with the innate immune system, especially represented by macrophages, dendritic cells (DCs) and neutrophils [12].

The family of toll like receptors (TLRs) are trans-membrane proteins expressed mainly in macrophages, DCs, natural killer (NK) cells and lymphocytes (T and B); they are specialized in mediating the innate recognition of pathogens associated molecules patterns (PAMPs), that are presented in a huge range of pathogens of clinical and immunological relevance [13] and rarely found in the host cells [14, 15]. Recognition of each PAMP appears to be associated to distinct TLRs. Once the response is set, the activation of specific signaling pathways [16] rapidly triggers a variety of phenomena that amplify parasite immune responses by stimulating the production of pro- inflammatory cytokines, which may play an important role in controlling *Leishmania* infection [17]. TLR2 was shown to recognize ligands such as lipopeptides [18], peptidoglycans [19] and external proteins [20], among others. As soon as TLR2 is linked to its ligands, the induction of intracellular pathways such as MyD88 activates nuclear factor (NF)-kB promoting the secretion of pro- and anti-inflammatory cytokines. Published studies that contribute to the knowledge of TLR2 in *Leishmania* infection are limited [21]. A protective role during infection was proposed for TLR2 as one of the molecules involved in *Leishmania* phagocytosis [22]. As another example, an experimental mouse model study using TLR2 agonist (Pam3CSK4) has demonstrated protection against *Leishmania* infection [23].

Currently, treatments are not always effective against the disease and a development of long-lasting vaccine would be a cornerstone in the prevention of disease. Therefore, it is important to discover new immunomodulators for prevention and treatment of this important canine zoonotic infectious disease. Based on previous published findings, the hypothesis of this study was that TLR2 agonist alone will enhance the production of inflammatory cytokines in canine ex vivo whole blood. In addition, we hypothesized that the combination TLR2 agonist with *L. infantum* soluble antigen might promote a synergistic release of pro-inflammatory cytokines when compared with *L. infantum* antigen or TLR2 ligand alone in previously *L. infantum-*infected dogs. Therefore, the main objective of this study was to investigate and compare the effect of a TLR2 agonist (TLR2a) alone or in combination with *L. infantum* antigen on ex vivo whole blood cytokines production from dogs in different stages of infection (seropositive sick, “resistant” (Ibizan hounds) and seronegative clinically healthy dogs).

## Methods

### Dogs and sampling

The dogs enrolled in the study were from different Catalonian and Balearic Islands veterinary centers in Spain and were divided into three groups: Group 1: 17 dogs with clinical leishmaniosis from Fundació Hospital Clínic Veterinari (Bellaterra, Barcelona) and Hospital Ars Veterinaria (Barcelona); Group 2: 21 healthy Ibizan hound from a highly endemic area of CanL (the Island of Mallorca, Spain) [5]; and Group 3: 11 clinically healthy dogs from a low-endemicity area [24] (Asturias, Spain) with no travel history outside Asturias. The diagnosis of CanL was made based on the results of the physical examination, a complete blood count using System Siemens Advia 120 (Siemens Healthcare GmbH, Germany), a biochemical profile including creatinine, urea, total proteins, ALT and total cholesterol measured by the Olympus Analyzer AU 400 (Olympus, Center Valley, USA), protein serum electrophoresis by Hydrasys® (Sebia Electrophoresis, Lisses, France), urinalysis with urinary protein/creatinine ratio (UPC) and quantitative serology for the detection of *L. infantum*-specific antibodies by means of a serial dilution in house ELISA [25]. Cytological evaluation of any lesion or cutaneous histology and/or immunohistochemistry for *Leishmania* was also performed as described elsewhere in some cases when needed [26]. In addition, blood DNA extraction and *L. infantum* real-time PCR (RT-PCR) were performed as previously described [25]. Dogs were classified in four clinical stages (I, mild disease; II, moderate disease; III, severe disease; and IV, very severe disease) at the time of diagnosis as previously described [2].

### Whole blood cytokine release assay

Heparinized whole blood cytokine release assay was performed as previously described [27] with some modifications. Briefly, five different treatment conditions were established: (i) medium alone (Ø); (ii) medium with soluble *L. infantum* antigen (LSA) at a concentration of 10 μg/ml provided by Dr. Cristina Riera (*L. infantum* antigen 5 mg/ml, Facultat de Farmacia, Universitat de Barcelona); (iii) medium with mitogen concanavalin A (ConA, 100 mg Medicago® Uppsala, Sweden) at a concentration of 10 μg/ml; (iv) medium with TLR2 receptor agonist (Ø + TLR2a) at a concentration of 300 ng/ml (Pam3CSK4 1 mg/mL Invivogen® San Diego, California); and (v) medium with TLR2 receptor agonist at concentration of 300 ng/ml and soluble *L. infantum* antigen (LSA+TLR2a) at a concentration of 10 μg/ml. The plates were incubated at 37 °C in 5% of CO_2_ air. Then, blood was centrifuged at 300× *g* for 10 min and the supernatant was collected and stored at −80 °C until used. TNF-α and IL-6 concentrations were measured in supernatants from 48 h. IFN-γ was measured in supernatants from 5 days after stimulation with ConA and LSA or medium alone as previously described [27].

### Sandwich ELISA for canine cytokines

Cytokine analysis of IFN-γ, TNF-α, and IL-6 was performed according to the manufacturer’s instructions (DuoSet® ELISA by Development System R&DTM, Abingdon, UK) using 96-well cell plate flat bottom (Costar ® Corning, NY, USA). Slight modifications were done for the IFN-γ ELISA as described elsewhere [27]. Standard curve for TNF-α started with 1000 pg/ml and two-fold dilutions were made until 7.8 pg/ml concentration. Finally, standard curve for IL-6 starting with 4000 pg/ml and two-fold dilutions were made until 31.2 pg/ml concentration. Each cytokine concentration for all treatment conditions studied was analyzed after substracting medium alone for comparison between groups. Dogs were classified as IFN-γ producers and non-producers as previously described [27].

### Statistical analysis

The statistical analysis was performed using the SPSS 22.0 software for Windows (SPSS Inc., USA). A non-parametric Mann-Whitney *U*-test was used to compare groups. A non-parametric Wilcoxon signed-rank test was used to compare paired continuous variables. Differences were considered significant with a 5% significance level (*P* < 0.05). Graphs were performed using excel GraphPad Prism 7 (GraphPad Software, La Jolla, CA, USA).

## Results

### Clinical data

All 11 clinically healthy dogs from a low-endemic area of CanL (Group 3) were seronegative. There were five females (three spayed and two intact) and six males (three neutered and three intact) with a median of age of 49 months and an age range from 17 months to 12 years. Seven were purebred and four mixed breed.

The median of age of 21 clinically healthy Ibizan hounds (Group 2) was 27 months with a range from seven months to five years. Four males and 17 females were studied. In addition, all Ibizan hounds studied were seronegative with the exception of two dogs that were low positive.

Dogs with clinical leishmaniosis (Group 1), five females and 12 males, were mainly purebred (*n* = 14) and only three were mixed breed. The median age at the time of diagnosis was 79 months with a range from eight months to 17 years. All sick dogs presented at the time of diagnosis several typical clinical signs of leishmaniosis. Dogs were classified in the following clinical stages [2, 28]: II-moderate disease (stage IIa, *n* = 4 and stage IIb, *n* = 3); III-severe disease (*n* = 7); and IV-very severe disease (*n* = 3).

### Antibody levels and parasite specific IFN-γ production in all groups studied

**Fig. 1:**
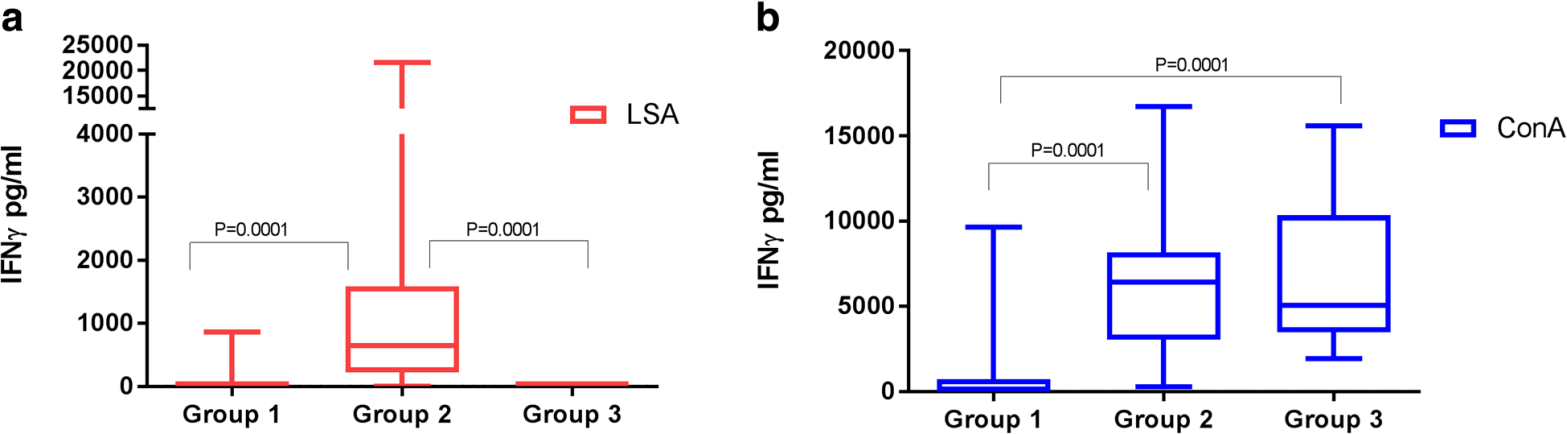
IFN-γ concentrations after whole blood stimulation with LSA and ConA in all groups studied. Sick dogs (Group 1), Ibizan hounds (Group 2) and healthy controls (Group 3). **a** LSA: Group 2 > Group 3 (Mann-Whitney *U-*test: *Z* = -4.15, *P* = 0.0001); Group 2 > Group 1 (Mann-Whitney *U-*test: *Z* = - 4.53, *P* = 0.0001). **b** ConA: Group 2 > Group 1 (Mann-Whitney *U*-test: *Z* = -4.15, *P* = 0.0001) and Group 3 > Group 1 (Mann-Whitney *U*-test: *Z* = -3.68, *P* = 0.0001)

The results of IFN-γ concentrations for each condition are shown in Fig. 1. Clinically healthy dogs from Asturias (Group 3: mean ± SD: 13.3 ± 5.7 EU; Mann-Whitney *U-*test: *Z* = -4.39, *P* = 0.0001) and Mallorca (Group 2: 16.6 ± 14.2 EU; Mann-Whitney *U-*test: *Z* = -5.24, *P* = 0.0001) presented statistically significant lower levels of antibodies than dogs with clinical leishmaniosis (Group 1: 22,747.1 ± 33,756.4 EU), respectively. No statistically significant differences were found when both clinically healthy groups were compared.

In general, healthy control dogs from the low endemicity area (Group 3) did not response to IFN-γ after LSA stimulation. Two out of 17 dogs with clinical leishmaniosis were classified as IFN-γ producers and further classified as being in stage IIa. The majority of the dogs were classified as IFN-γ non-producers (88%). The clinical staging of these dogs was: two dogs in stage IIa (13.3%), two dogs in stage IIb (13.3%), eight dogs in stage III (53.3%) and three dogs in stage IV (20%). Only two Ibizan hounds did not respond to IFN-γ after LSA stimulation, the rest of the dogs (90%) responded at high levels.

### Whole blood TNF-α release assay

**Fig. 2:**
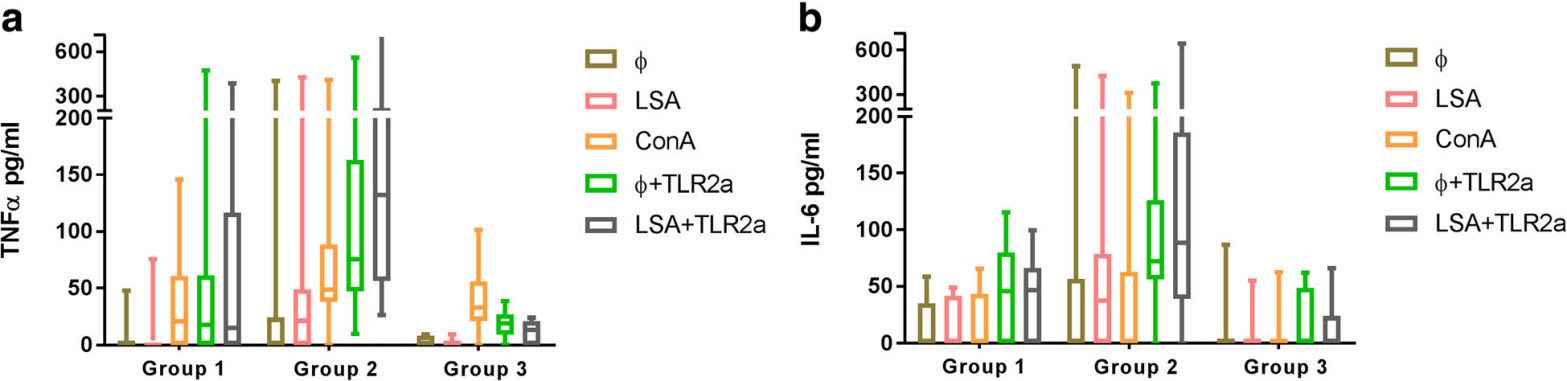
**a** TNF-α and **b** IL-6 concentrations from the three groups of dogs studied after each condition. Sick dogs (Group 1), Ibizan hounds (Group 2) and healthy controls (Group 3). An additional file shows statistics (see Additional file 1). Panels: Medium alone (Ø), soluble *L*. *infantum* antigen (LSA), concanavalin A (ConA), TLR2 (Pam3CSK4) receptor agonist (Ø+TLR2a) and TLR2a and LSA (LSA+TLR2a)

The results of TNF-α concentration from the three groups of dogs studied for each condition are shown in Fig. 2a and Additional file 1. An additional file shows statistical differences (see Additional file 1). The most important finding was the significant higher production of TNF-α after stimulation with ConA, Ø + TLR2a, LSA + TLR2a when compared with medium alone in the three groups studied. Interestingly, only Ibizan hounds produced significantly higher levels of TNF-α after stimulation with LSA when compared with medium alone. Furthermore, Ø + TLR2a and LSA + TLR2a elicited higher production of TNF-α than LSA alone in all three groups studied. No significant differences were found in TNF-α concentrations when comparing Ø + TLR2a stimulated blood and LSA + TLR2a in sick (Group 1) and control (Group 3) dogs while stimulated blood from Ibizan hounds (Group 2) showed statistically significant lower TNF-α production on Ø + TLR2a when compared with LSA + TLR2a.

No significant differences were found when control healthy dogs of Group 3 were compared with sick dogs in all conditions studied. In contrast, Ibizan hounds (Group 2) secreted significantly higher levels of TNF-α than control dogs (Group 3) and sick dogs (Group 1) after Ø + TLR2a and LSA + TLR2a.

### Whole blood IL-6 release assay

The results of IL-6 concentration in all groups studied after each condition are shown in Fig. 2b and Additional file 1. An additional file shows statistical differences (see Additional file 1). The healthy control (Group 3) did not present any significant differences within treatments in IL-6 secretion. The sick dogs (Group 1) and Ibizan hounds (Group 2) presented significantly lower concentrations of IL-6 in medium alone when compared with Ø + TLR2a and LSA + TLR2a stimulation. In addition, Ø + TLR2a and LSA + TLR2a elicited significantly higher stimulation of IL-6 than LSA alone in Groups 1 and 2. There was no statistically significant difference in IL-6 production within Ø + TLR2a and LSA + TLR2a in the sick dogs (Group 1) and Ibizan hounds (Group 2). Ibizan hounds (Group 2) secreted significantly higher levels of IL-6 than control dogs (Group 3) and sick dogs (Group 1), after Ø + TLR2a and LSA + TLR2a.

## Discussion

Pam3CSK4 is a synthetic derivative of triacylated lipoproteins that conserves most of the immune stimulatory activity of full-length lipoproteins [29]. Here, we explored the impact of the lipopeptide Pam3CSK4 in whole blood from dogs. To the best of our knowledge, the findings of the present study give new insights, for the first time, on the inflammatory effects that, the Pam3CSK4 TLR2 agonist alone or in combination with *L. infantum* antigen, induce in ex vivo whole blood dogs in different stages of *Leishmania* infection (sick, “resistant” and non-infected healthy dogs).

Our findings demonstrate that the Pam3CSK4 TLR2 agonist alone significantly increased the production of TNF-α as previously described [30, 31]. In agreement with the present study, stimulation of purified canine polymorphmononuclear cells (PMNs) with lipoteichoic acid, a ligand of TLR2, promoted the release of pro-inflammatory chemokine IL-8 [32]. In this study, the Pam3CSK4 TLR2 agonist alone also significantly increased the production of IL-6. The main cell sources of cytokine production after stimulation with a TLR2 agonist in the present study are likely to be granulocytes and monocytes due to the fact that granulocyte is the predominant inflammatory nucleated cell in whole blood in canines [33]. In addition, it has been also demonstrated that TLR2 protein is easily detectable by flow cytometry on the canine peripheral blood granulocyte and monocyte cell surfaces and less strongly in lymphocytes [32]. This is similar to findings in humans where lymphocytes do not express TLR2 in unstimulated blood [34]. This study corroborates the pro-inflammatory effect that the Pam3CSK4 TLR2 agonist has in canines.

A significant strong activation of a pro-inflammatory response was observed in dogs studied with high levels of TNF-α and IL-6 after TLR2a blood stimulation. However, significantly stronger TNF-α and IL-6 responses after TLR2a blood stimulation were observed in Ibizan hounds when compared with sick and control dogs. The relation of TLR2 and TLR4 in pro- and anti-inflammatory cytokine production was previously investigated in human patients with visceral leishmaniasis (VL). Gatto et al. [35] stimulated PBMCs from cases of VL with TLR2 and TLR4 agonists and later cytokine production and nitric oxide (NO) was evaluated. In agreement with the present study, they also described higher levels of TNF-α in patients with visceral leishmaniosis after stimulation with TLR2 or TLR4 agonists [35]. In addition, analysis of the involvement of TLR2 and TLR4 agonists in NO production demonstrated that these two receptors appeared to be involved in NO production.

Interestingly, a significant increase of TNF-α production was observed when whole blood from Ibizan hounds was stimulated with a combination of *L. infantum* antigen and TLR2 agonist when compared with *L. infantum* antigen alone and TLR2 agonist alone suggesting a synergistic pro-inflammatory effect. It is likely that this synergistic pro-inflammatory effect is due to the TNF-α release by granulocytes and monocytes and to less extent to activated or memory T lymphocytes as described in humans [36]. TLR2 is expressed in human activated T cells as a costimulatory receptor and memory T cells [36]. Thus, human TLR2 serves as a costimulatory receptor for antigen-specific T cell development and participates in the maintenance of T cell memory and it is likely that the same process occurs in canines. This suggests that pathogens, *via* their pathogen-associated molecular patterns, may contribute directly to the perpetuation and activation of long-term T cell memory in both antigen-dependent and independent manners. These findings are similar to the ones documented by combinations of TLR4 or TLR7 agonists and vaccine antigens leading to a more robust Th1 CD4+ T cell responses from sub-clinically infected dogs [37]. Therefore, based on the present findings, Pam3CSK4 TLR2 agonist might be used as an adjuvant in future vaccine development having an impact on controlling this infection.

Furthermore, although the data presented here result from an ex-vivo study in dogs and murine models as well as *L. donovani* or *L. major* strains are very different from canine *L. infantum* infection [38], when susceptible and “resistant” mice were immunized with live *L. major* in the presence of Pam3CSK4 (TLR2 agonist), it was found that the development of skin lesion in both groups of animals were prevented but at different magnitudes [23]. Once again in another murine study, TLR2 seemed to have an active role in the control of cutaneous leishmaniosis since TLR2-deficient mice presented an exacerbation of the pathology and parasitemia through promotion of the Th2 immunity in *L. major* and *Leishmania mexicana* infections [39]. However, it is important to highlight that TLR2/6 ligand Pam2CSK4 is a Th2 polarizing adjuvant in *L. major* and *Brugia malayi* murine vaccine models [40]. In addition, in a canine study, a L111f vaccine antigen containing a LeIF, a TLR2 agonist glycoprotein produced Th2 skewed responses leading to less robust CD4+ T cell population responses [37]. Further research needs to be carried out to better elucidate the findings of this study. It is important to highlight that activation of TLR receptors by specific agonists as in the case of Pam3CSK4 TLR2 agonist could be a powerful tool in the control and treatment of the CanL, either as adjuvant in future vaccine development or during treatment as immunomodulator to control infection in sick dogs. The use of TLR2 agonist in combination with conventional treatment (meglumine antimoniate or miltefosine + allopurinol) [2] might allow reduction of the anti-*Leishmania* drug dose or shortening the length of conventional treatment avoiding long term side effects [41] and drug resistance [42].

Ibizan hounds are considered a more “resistant” breed to *L. infantum* infection due to the presence of a protective immune response that is associated with a clinically healthy status and good outcome [2, 5, 43]. Data presented here showed a marked IFN-γ response to LSA after blood stimulation in Ibizan hounds when compared with control and sick dog groups. In this study, an overall seronegative result seen in Ibizan hounds in combination with a high production of specific *L. infantum* IFN-γ and TNF-α corroborates the previous findings demonstrating a predominance of *L. infantum* specific cellular immunity by means of leishmanin skin test in Ibizan hounds living in a highly endemic area of leishmaniosis [5].

Taking these findings into account, the Ibizan hound is an excellent canine breed model for the study of the protective anti-*Leishmania* immune response and for comparison to sick and control healthy dogs in endemic areas as well as to other “resistant” animal models.

## Conclusions

This study demonstrated that TLR2a alone enhances the production of the inflammatory cytokines TNF-α and IL-6 in sick, “resistant” and healthy non-infected dogs. In addition, a combination of LSA+TLR2a promoted a synergistic pro-inflammatory effect with TNF-α in Ibizan hounds but not in seropositive sick dogs and seronegative healthy dogs. These findings might suggest the importance of Pam3CSK4 as a possible immunomodulator for CanL either as an adjuvant for a future vaccine development or as immunotherapy in dogs with clinical illness.

## Additional file

Additional file 1: Statistical differences in TNF-α and IL-6 concentrations for conditions and among groups studied. (DOC 28 kb)

## Abbreviations

(NF)-kB: Nuclear factor kB
CanL: Canine leishmaniosis
CD4: Cluster of diferentiation
CMI: Cell mediated immunity
ConA: Concanavalin A
DC: Dendritic cell
ELISA: Enzyme-linked immunosorbent assay
EU: ELISA units
IFN-γ: Interferon-gamma
IL-2: Interleukin-2
IL-6: Interleukin-6
IL-8: Interleukin-8
LSA: *Leishmania infantum* soluble antigen
NK: Natural killer
NO: Nitric oxide
PAMP: Pathogen-associated molecular patterns
PBMC: Peripheral blood mononuclear cells
PD-1: Programmed cell death protein 1
PMNs: Purified canine polymorphmononuclear cells
ROS: Reactive oxygen substances
rt-PCR: Real time PCR
Th1: Type 1 T helper cells
Th2: Type 2 T helper cells
TLR: Toll like receptor
TLR2: Toll like receptor 2
TLR2a: Toll like receptor 2 agonist
TLR4: Toll like receptor 4
TNF-α: Tumor necrosis factor-alpha
VL: Visceral leishmaniosis
