# Supplementary material for: The inflammatory cytokine effect of Pam3CSK4 TLR2 agonist alone or in combination with Leishmania infantum antigen on ex-vivo whole blood from sick and resistant dogs
Source: Parasit Vectors. 2017 Mar 13;10:123. doi: 10.1186/s13071-017-2062-3 (PMC5346840; doi:10.1186/s13071-017-2062-3)
Supplement: Additional file 1: — Statistical differences in TNF-α and IL-6 concentrations for conditions and among groups studied. (DOC 28 kb) [file 13071_2017_2062_MOESM1_ESM.doc]

TNF-α in each group of dogs studied:

• Group 1: ConA > Ø (Wilcoxon signed-rank test: *Z* = -2.22, *P* = 0.026), Ø+TLR2a > Ø (Wilcoxon signed-rank test: *Z* = -2.59, *P* = 0.009) and LSA+TLR2a > Ø (Wilcoxon signed-rank test: *Z* = -2.93, *P* = 0.003), Ø+TLR2a > LSA (Wilcoxon signed-rank test: *Z* = -2.39, *P* = 0.017) and LSA+TLR2a > LSA (Wilcoxon signed-rank test: *Z* = -2.93, *P* = 0.003)

• Group 2: ConA > Ø (Wilcoxon signed-rank test: *Z* = -3.32, *P* = 0.001), LSA > Ø (Wilcoxon signed-rank test: *Z* = -1.99, *P* = 0.046), Ø+TLR2a > Ø (Wilcoxon signed-rank test: *Z* = -4.01, *P* = 0.0001), LSA+TLR2a > Ø (Wilcoxon signed-rank test: *Z* = -4.01, *P* = 0.0001), Ø+TLR2a > LSA (Wilcoxon test: *Z* = -3.42, *P* = 0.001) and LSA+TLR2a > LSA (Wilcoxon signed-rank test: *Z* = -3.58, *P* = 0.0001), LSA+TLR2a > Ø+TLR2a (Wilcoxon signed-rank test: *Z* = -3.21, *P* = 0.001).

• Groups 3: ConA > Ø (Wilcoxon signed-rank test: *Z* = -2.80, *P* = 0.005), Ø+TLR2a > Ø (Wilcoxon signed-rank test: *Z* = -2.66, *P* = 0.008), LSA+TLR2a > Ø (Wilcoxon signed-rank test: *Z* = -2.36, *P* = 0.018), LSA+TLR2a > LSA (Wilcoxon signed-rank test: *Z* = -2.36, *P* = 0.018), ConA > LSA+TLR2a (Wilcoxon signed-rank test: *Z* = -2.59, *P* = 0.009).

TNF-α among groups and conditions:

Ø+TLR2a: Group2 > Group 3 (Mann-Whitney U-test: *Z* = -3.94, *P* = 0.0001) and Group2 > Group1 (Mann-Whitney U-test: *Z* = -2.75, *P* = 0.005)

LSA+TLR2a: Group2 > Group 3 (Mann-Whitney U-test: *Z* = -4.22, *P* = 0.0001) and Group 2 > Group 1 (Mann-Whitney U-test: *Z* = -2.75, *P* = 0.005)

IL-6 in each group of dogs studied:

• Group 1: ConA > Ø (Wilcoxon signed-rank test: *Z* = -2.38, *P* = 0.017), Ø+TLR2a > Ø (Wilcoxon signed-rank test: *Z* = -2.80, *P* = 0.005), LSA+TLR2a > Ø (Wilcoxon signed-rank test: *Z* = -3.059, *P* = 0.002), Ø+TLR2a > ConA (Wilcoxon signed-rank test: *Z* = -2.13, *P* = 0.033), LSA+TLR2a > ConA (Wilcoxon signed-rank test: *Z* = -2.98, *P* = 0.003). Ø+TLR2a>LSA (Wilcoxon signed-rank test: *Z* = -2.80, P = 0.005) and LSA+TLR2a>LSA (Wilcoxon signed-rank test: *Z* = -3.05, *P* = 0.002)

• Group 2: Ø+TLR2a > Ø (Wilcoxon signed-rank test: *Z* = -2.27, *P* = 0.023), LSA+TLR2a > Ø (Wilcoxon signed-rank test: *Z* = -2.38, *P* = 0.017), Ø+TLR2a > LSA (Wilcoxon signed-rank test: *Z* = -2.35, *P* = 0.019), LSA+TLR2a>LSA (Wilcoxon signed-rank test: *Z* = -2.10, *P* = 0.035)

IL-6 among groups and conditions:

Ø+TLR2a: Group 2 > Group 3 (Mann-Whitney U-test: *Z* = -3.07, *P* = 0.002) and Group 2 > Group 1 (Mann-Whitney U-test: *Z* = -2.09, *P* = 0.039)

LSA+TLR2a: Group 2 > Group 3 (Mann-Whitney U-test: *Z* =-2.97, *P* = 0.003) and Group 2 > Group 1 (Mann-Whitney U-test: *Z* = -2.22, *P* = 0.026)
